# *“Spoiled”* girls: Understanding social influences on adolescent contraceptive decision-making in Kenya

**DOI:** 10.1371/journal.pone.0255954

**Published:** 2021-08-12

**Authors:** Elizabeth K. Harrington, Edinah Casmir, Peninah Kithao, John Kinuthia, Grace John-Stewart, Alison L. Drake, Jennifer A. Unger, Kenneth Ngure

**Affiliations:** 1 Department of Obstetrics & Gynecology, University of Washington, Seattle, Washington, United States of America; 2 Kenya Medical Research Institute, Center for Clinical Research, Thika, Kenya; 3 Department of Research & Programs, Kenyatta National Hospital, Nairobi, Kenya; 4 Department of Global Health, University of Washington, Seattle, Washington, United States of America; 5 Department of Medicine, University of Washington, Seattle, Washington, United States of America; 6 Department of Epidemiology, University of Washington, Seattle, Washington, United States of America; 7 Department of Pediatrics, University of Washington, Seattle, Washington, United States of America; 8 Department of Community Health, Jomo Kenyatta University of Agriculture and Technology, Nairobi, Kenya; University of Salamanca, SPAIN

## Abstract

**Objectives:**

Despite significant public health emphasis on unintended pregnancy prevention among adolescent girls and young women in Sub-Saharan Africa, there is a gap in understanding how adolescents’ own reproductive priorities and the social influences on their decision-making align and compete. We examined the social context of contraceptive decision-making among Kenyan female adolescents.

**Methods:**

Using community-based sampling, we conducted 40 in-depth interviews and 6 focus group discussions among sexually-active or partnered adolescent girls and young women aged 15–19 in the Nyanza region of Kenya. We analyzed the data in Dedoose using an inductive, grounded theory approach, and developed a conceptual model from the data illustrating social influences on adolescent contraceptive decision-making.

**Results:**

Participants viewed adolescent pregnancy as unacceptable, and described severe social, financial, and health consequences of unintended pregnancy, including abortion under unsafe conditions. Yet, their contraceptive behaviors often did not reflect their desire to delay pregnancy. Contraceptive decision-making was influenced by multiple social factors, centering on the intersecting stigmas of adolescent female sexuality, pregnancy, and contraceptive use, as well as unequal power in sexual relationships. To prioritize pregnancy prevention, adolescents must navigate conflicting social norms and power dynamics, and put their perceived future fertility at risk.

**Conclusions:**

Contraceptive decision-making among Kenyan female adolescents is strongly influenced by opposing social norms within families, communities, and sexual relationships, which compel them to risk stigma whether they use a contraceptive method or become pregnant as adolescents. These findings put into perspective adolescents’ seemingly incongruent pregnancy preferences and contraceptive behaviors. Interventions to address adolescent unintended pregnancy should focus on supporting adolescent decision-making agency, addressing fertility-related contraceptive concerns, and promoting innovative contraceptive access points rather than increasing contraceptive prevalence.

## 1. Introduction

Adolescent girls and young women aged 15–19 living in Sub-Saharan Africa are underserved by existing sexual and reproductive health (SRH) service delivery [1]. Research consistently demonstrates adolescents’ disproportionate risk of HIV acquisition and unintended pregnancy [2, 3], and the related poor outcomes of maternal morbidity and mortality and complications of abortion under unsafe conditions [4, 5]. Forty-five percent of pregnancies among African adolescents are estimated to be unintended (63% in Kenya [6]), and nearly two-thirds are classified as having unmet need for contraception—meaning they are sexually active and report not wanting a child for at least 2 years, but are not using a contraceptive method [3]. In response to these indicators and the unique developmental and social circumstances facing adolescents, researchers, governments, and funders have advocated for a renewed emphasis on improving adolescent SRH [7]. With the goal of promoting adolescent SRH, the global public health discourse endorses targeting this population with interventions to increase contraceptive prevalence—or reduce unmet need—through voluntary and tailored family planning services [8].

Despite substantial investments in adolescent SRH and high contraceptive availability in many countries [8, 9], however, contraceptive prevalence has been slow to rise among adolescents [10]. Multiple sociocultural and health systems barriers intersect to influence adolescents’ contraceptive access and use [11]. Adolescent sexuality and contraceptive use are highly stigmatized in many communities [12–14], and discriminatory treatment from healthcare providers further deters SRH care-seeking [15, 16]. Adolescents are susceptible to misconceptions about contraceptive risks, especially future fertility concerns, which are rooted in gender and social norms [17–19].

Furthermore, it is clear that unmet need for contraception—a frequently-used concept and indicator referring to fecund women whose stated desire is to delay or limit childbearing but are not using a contraceptive method [20]—does not equate with desire to use contraception [21]. The public health research and policy narratives have focused on the former, leading to a gap in knowledge about how adolescents in sub-Saharan Africa who are most likely to experience unintended pregnancy perceive their own pregnancy risk and contraceptive need, or make decisions about contraception within their lived realities and social networks. Thus, the reproductive priorities of adolescents classified as having an unmet need for contraception, and influences on their contraceptive use or nonuse, have not been adequately characterized. These needs and drivers of SRH must be framed by the complex and often non-binary nature of reproductive preferences. A growing body of literature has highlighted the limits of using the pregnancy planning paradigm, or dichotomous categories of planned/unplanned and intended/unintended, to understand contraceptive needs [22]. More reproductive preference data and measures specific to African contexts and adolescent populations are needed to guide interventions to support contraceptive decision-making. These research gaps are particularly germane to adolescents younger than 18; younger, unmarried adolescents are underrepresented in SRH research due to ethical constraints and parental consent requirements, though studies suggest they are even more vulnerable to poor health outcomes [23, 24].

This study explores how Kenyan adolescent girls and young women aged 15–19 perceive their own contraceptive needs and make contraceptive decisions within their social contexts. We examine the social influences on contraceptive decision-making among adolescents at risk for pregnancy, aiming to provide nuanced insights into adolescents’ contraceptive behaviors. Our research questions and approach were guided by constructs of Social Cognitive Theory [25] and Social Norms Theory [26]. Specifically, we focused on conceptualizing the dynamic interplay between adolescent behavior and their social environment, and adolescents’ perceived self-efficacy in making contraceptive decisions [27]. We drew on insights of literature exploring how social norms affect reproductive health behaviors [26, 28, 29]. Through advancing understandings of adolescent contraceptive decision-making, this study aims to inform adolescent-centered efforts to better align reproductive preferences and outcomes for this underserved population.

## 2. Materials and methods

### 2.1 Study design and setting

For this qualitative study, we selected in-depth interview (IDI) and focus group discussion (FGD) methods to provide data source triangulation, maximizing the benefits of IDIs for gathering data on complex and sensitive topics, and FGDs for examining questions within the context of social interaction [30]. We conducted 40 IDIs and 6 FGDs with adolescents aged 15–19 in the Nyanza region of Kenya between October 2017-March 2018. This region, which has the highest HIV burden in Kenya and high relative unmet need for contraception [31], was selected due to existing research infrastructure in the area. Study activities took place in the peri-urban Ahero community and the primarily rural area near Bondo town in Kisumu and Siaya counties, respectively. Luo is the primary ethnic group and language of the Nyanza region. All authors have had long-term research engagement in women’s reproductive health research in the study region and have extensive collective experience working with adolescent populations in Kenya.

### 2.2 Sampling and eligibility

Prior to initiating community-based recruitment, a community advisory board was consulted to optimize communication about the study with prospective participants, parents of adolescents, and other community members. Our all-female data collection team consisted of two local qualitative interviewers with backgrounds in social science, assisted by two community mobilizers. The principal investigators (PIs; EH and KN) trained the team, emphasizing ethical conduct of sensitive research among adolescents. The semi-structured guides were piloted, revised, and collaboratively translated to optimize question comprehension. The team worked alongside community health volunteers to recruit participants for IDIs or FGDs in various community venues, including markets, private homes, and youth gatherings. Using purposive and snowball sampling strategies, the study team recruited in rural and peri-urban locations in Kisumu and Siaya counties. Focus groups were disaggregated by age group (14–17 and 18–19) to optimize group dynamics.

Adolescents were eligible to participate if they were between the ages of 14–19; had a parent/guardian willing provide consent (if <18); unmarried; spoke English, Luo, or Swahili; and were “at risk” for pregnancy. Adolescents who had ever been sexually active or used a family planning method, or were in a current romantic relationship with a male partner regardless of sexual activity, were considered “at risk.” We excluded adolescents with a prior live birth in order to focus on adolescents with less representation in existing literature, but not those who had experienced spontaneous or induced abortion.

Adolescents were approached in the community, and eligibility assessed privately by a study staff member. For adolescents 18 or older, written informed consent was signed. Written parental consent and adolescent assent were obtained for participants under 18. Participants received 400 Kenyan Shillings (approximately USD$4) to ensure transportation cost coverage. This study was approved by the Kenyatta National Hospital Ethics and Research Committee, the Human Subjects Division at the University of Washington, and the County Health Management Teams in Kisumu and Siaya counties.

### 2.3 Data collection

**Fig 1:**
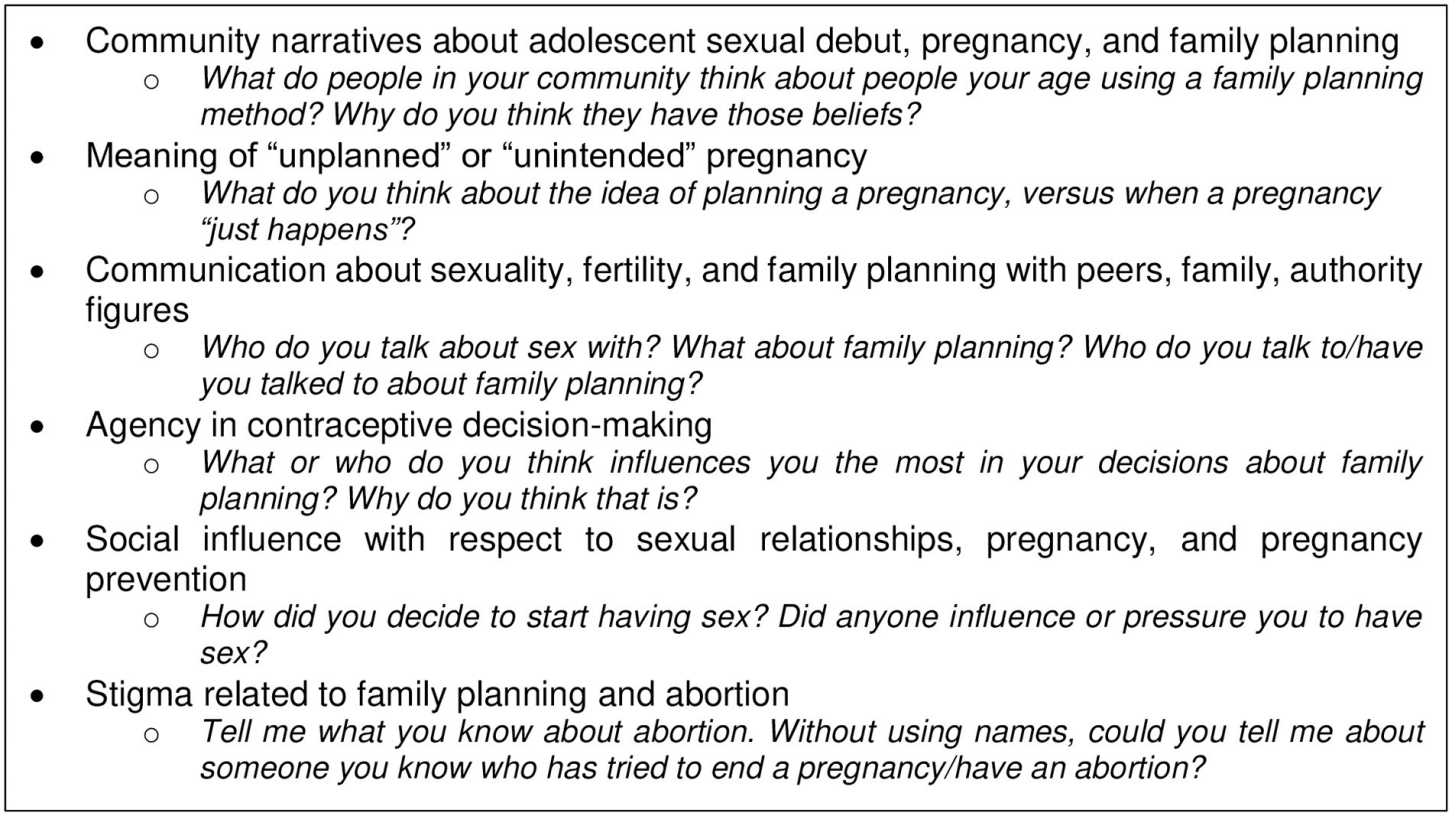
Primary in-depth interview/focus group discussion domains and sample questions.

The primary IDI/FGD domains represented in the semi-structured interview guide are listed in Fig 1. Each participant completed a brief tablet-based socio-demographic questionnaire, which was administered verbally by study staff. IDIs were primarily conducted in private homes; when privacy could not be assured or when preferred by the participant, study offices located at two county health facilities were used. FGDs were held with permission in private areas of county health facilities. Staff were fluent in written and spoken English, Luo, and Kiswahili; IDIs and FGDs were conducted in the language preferred by participants. IDIs and FGDs were digitally audio-recorded and simultaneously transcribed and translated into English by the interviewers themselves; a different staff member reviewed each transcript for accuracy of transcription and translation.

### 2.4 Data analysis

**Fig 2:**
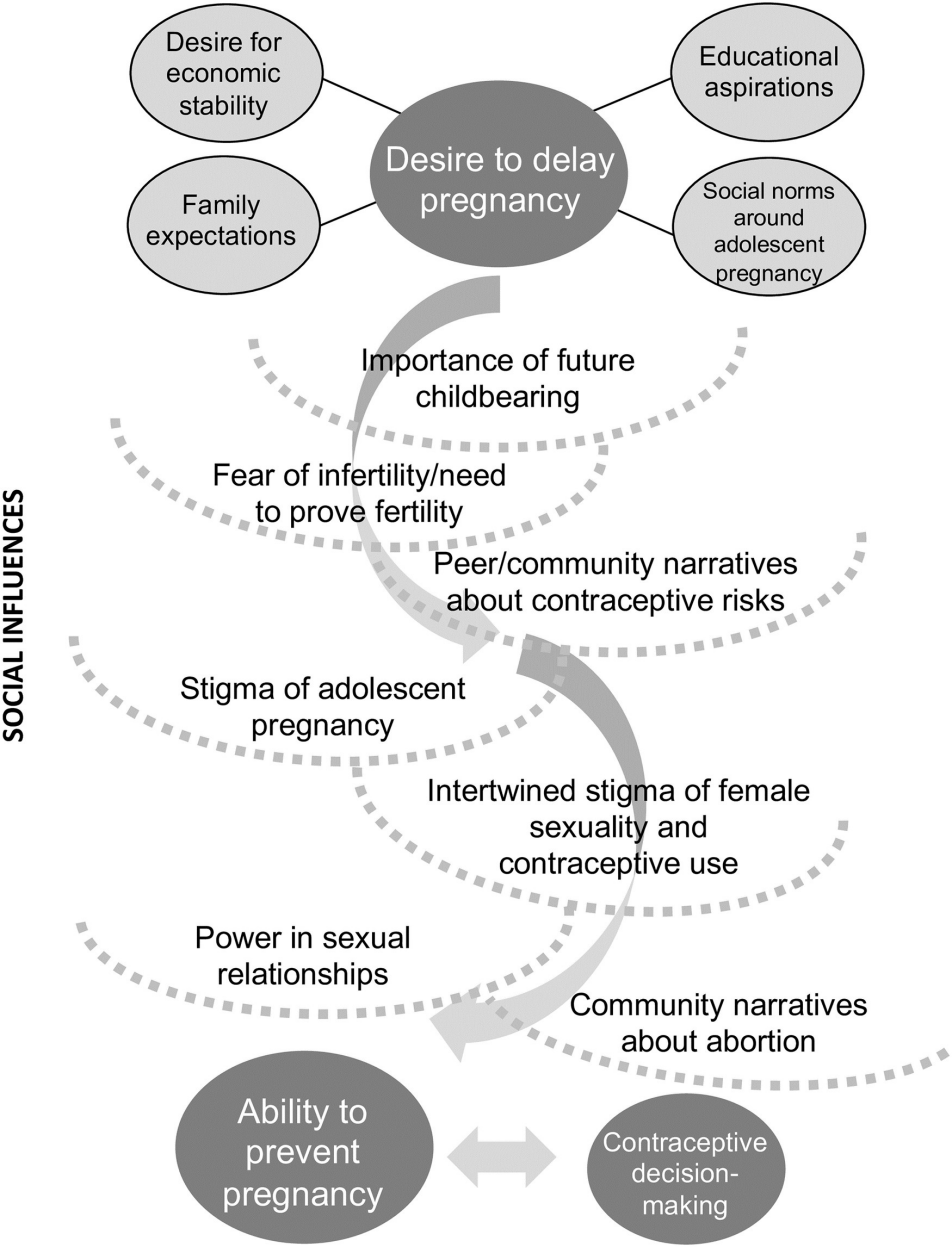
Conceptual model: Social influences on adolescent contraceptive decision-making.

We used an inductive, thematic approach to analyzing the qualitative data. Our iterative analytic strategy drew on contemporary grounded theory methods, described by Charmaz as “systematic, yet flexible guidelines” for qualitative analysis and theory-building [32]. The co-PIs agreed on a set of initial codes designed to reflect *a priori* domains of interest from the interview guides and exposure to the raw transcripts and field notes. The lead author (EH), who has over 10 years of experience working in Kenya, and a Kenya-based expert qualitative data analyst (EC) coded 5 IDIs in parallel, after which they compared code application and meaning, adding new codes as needed to identify emerging themes. After a second round of parallel coding, investigators constructed a final codebook for both IDIs and FGDs, and re-coded or coded all remaining transcripts. Two-thirds of transcripts were double-coded, and investigators met regularly to discuss coding discrepancies and emerging themes. The analytic team identified the most significant codes, grouped similar and contrasting excerpts within and between codes, and wrote analytic summaries for each major theme. Investigators conducted all coding in Dedoose (Version 8.0.35, SocioCultural Research Consultants, LLC, www.dedoose.com), an online qualitative analysis program facilitating data management and collaborative coding. The conceptual model (Fig 2) was developed during the analytic process in order to organize and interpret themes emerging from the data.

## 3. Results

Study staff screened 200 adolescent girls and young women in community settings, of whom 115 were eligible and 86 (75%) were enrolled: 40 in IDIs and 46 in FGDs. The median age was 17, with 11 (13%) participants aged 15 or younger (Table 1). The majority were currently students (71%), romantically partnered (81%), and reported ever being sexually active with a male partner (87%). Most (79%) reported ever using a contraceptive method; 71% of these had used male condoms alone. No participants reported a prior pregnancy.

**Table 1:** Participant characteristics.

Characteristic	Total n = 86 (except where noted) n(%); median (IQR)
Age (n = 85)	17 (16–18)
• *Age 14–15*	11 (13%)
• *Age 16–17*	38 (44%)
• *Age 18–19*	36 (42%)
• *Missing*	1 (1%)
County	
• Kisumu	42 (49%)
• Siaya	44 (51%)
Currently a student	61 (71%)
Age when quit school (n = 22)	18 (16–19)
Highest educational attainment	
• *Less than primary school*	10 (12%)
• *Primary school completed*	59 (71%)
• *Secondary school completed*	12 (15%)
• *Some post-secondary school*	2 (2%)
Currently in romantic relationship	70 (81%)
Length of relationship, years (n = 70)	1 (0.5–2)
Partner age (n = 68)	20 (18–22); min 13, max 30
Ever sexually active with male partner	75 (87%)
Age at sexual debut (n = 75)	15 (14–17); min 11, max 18
Lifetime sexual partners (n = 67)	1 (1–2); min 1, max 5
Ever pregnant (n = 78)	0
Ever tested for HIV (n = 78)	71 (91%)
Ever use, contraceptive method (n = 78)	68 (79%)
Ever method use (n = 68)	
• *Condoms as only method*	48 (71%)
• *Contraceptive implant*	10 (15%)
• *Depot medroxyprogesterone acetate*	5 (7%)
• *Emergency contraception*	2 (3%)
• *Oral contraceptives*	1 (1%)
• *Intrauterine contraception*	1 (1%)
• *Withdrawal*	1 (1%)
• *Condoms and another method*	12 (18%)
Current use, contraceptive method (n = 68)	35 (51%)
• *Condoms only*	23 (34%)
• *Contraceptive implant*	8 (24%)
• *Depot medroxyprogesterone acetate*	2 (3%)
• *Oral contraceptive*	1 (1%)
• *Withdrawal*	1 (1%)
• *Condoms and another method*	26 (38%)
Had sex in the last month (n = 60)	32 (53%)

Our findings synthesize the complex social factors at play as adolescents navigate pregnancy risk and decisions around contraceptive use. In the results and conceptual model (Fig 2) below, we present the primary themes emerging from the data: adolescent pregnancy intentions and consequences, the apparent incongruence between pregnancy desires and contraceptive behaviors, and the major social influences on adolescent contraceptive decision-making, including various sources of stigma.

### 3.1 Adolescent pregnancy: Intentions and consequences

Understanding adolescents’ pregnancy preferences is vital to gaining insight into their contraceptive decision-making. Respondents universally placed high value on future pregnancy and childbearing. The majority of girls resonated less with the concept of “planning” a pregnancy, and more with the idea that pregnancy is a “blessing” when it occurs at an ideal time, for example when education is complete and finances are stable. Participants viewed pregnancy while still in school or prior to financial independence extremely negatively. Many adolescents closely related adolescent pregnancy with lack of future educational attainment, which they explained led to living in poverty. Pregnancy among adolescents was seen as a burden to those close to them, and a challenge to meeting their basic needs for food and shelter. Speaking of a friend who had become pregnant, a young woman explained,

*“It was not a good life…it was a bad life because*, *you just see that grandmother has a problem*, *because she doesn’t have a job and she also has to care for the baby and the mother*, *so it could force her to go to the rice fields…It was very sad for me [to see her get pregnant]…because she was very young*, *so I was thinking*, *now this girl*, *she is young and she is pregnant*, *how will she survive*, *and how will her delivery be*, *and she doesn’t even have a mother*, *how will her baby survive?”*

—*IDI participant*, *Kisumu*, *age 19*

When participants were asked to share stories of adolescents in their communities who had become pregnant, they described multiple situations in rich detail, requiring very little prompting from interviewers. They emphasized the bleak social realities pregnant adolescents face: shame of dishonoring parents, community and peer stigma, and social withdrawal.

*“She was 13 years old and she wanted to remove [have an abortion] and the boyfriend also denied the pregnancy*, *but I just advised her that removing it can kill you*, *so she just gave birth… She used to be stressed up because she felt like removing*, *and the friends are also talking badly*, *so she never used to walk*, *she just kept sleeping in the house*. *She even thought of committing suicide but I just advised her until she gave birth to the baby*.*”*

—*IDI participant*, *Kisumu*, *age 17*

Considering how pregnancy now would affect their lives, many respondents focused on educational consequences. Parents’ and sponsors’ investments in school fees and financial support would be wasted. One participant (IDI participant, Kisumu, age 17) remembered her mother telling her pregnant sister, *“it is better to stop educating such a person*, *this is just wasting money for nothing*.*”* Another participant imagined social isolation of life-and-death magnitude:

*“I think if I become pregnant*, *I can’t carry it*, *because if I keep it…[I] am dead*. *My father can beat me to death…My schooling can just come to an end…so I think if I get pregnant I can just remove [have an abortion]*. *Even if I don’t remove*, *I don’t think I can stay alive*.*”*

—*IDI participant*, *Kisumu*, *age 16*

Very few adolescents expressed ambivalence about pregnancy preferences. However, reactions to potential unintended pregnancy were extremely varied. For example, a 17 year-old participant expressed a strong preference to wait until she had finished school to become pregnant. However, when asked about how she would feel about becoming pregnant now, she imagined a relatively casual, unconcerned reaction, contrasting the temporary nature of pregnancy with the permanence of HIV infection:

*“If I were to get pregnant now*, *I would just take it easy*, *befriend the nurses here [at the clinic]…I would be happy because pregnancy is not a disease that infects the body [like HIV]*, *you only carry it for 9 months and you deliver and you go back to your old self*.*”*

—*IDI participant*, *Kisumu*

### 3.2 Incongruent desires? Desire to avoid pregnancy and contraceptive behavior

All participants were previously or currently sexually active with a male partner or currently in a romantic relationship. While 20% of ever sexually-active adolescents declined to answer questions about frequency of sexual activity, 53% of those who responded reported sexual activity in the last month. Despite their sexual activity and their universally strong desire to avoid pregnancy while financially dependent on parents, hardly half (51%) of participants were using a contraceptive method; the majority of these were using condoms.

Many participants stated that they would have an abortion rather than continuing a pregnancy at their current life stage. This was striking to the authors in the context of participants’ broader narratives around abortion, which centered on numerous stories of peers and community members who experienced severe morbidity or death as a result of abortion under unsafe conditions. A notable, but not exceptional, example of this is a 17 year-old participant who recounted the story of her older sister, who had successfully aborted her pregnancy by drinking a local cola with tea leaves. The sister bled heavily, was eventually taken to the hospital, and survived. The participant then went on to talk about two classmates who had mixed laundry powder with water treatment concentrate and bleach to abort; both had died. She replied to the interviewer that *“If I become pregnant now…in my mind I am thinking that I can abort it because I want to first study so that I can get a baby later*.*”* This respondent, a 17-year-old IDI participant in Kisumu, was using a contraceptive implant. However, the majority of those who said they would have an abortion rather than continue an unintended pregnancy in adolescence, often specifically acknowledging risk of death or serious injury due to unsafe abortion, reported using condoms inconsistently in the absence of other method use.

Despite participants’ pregnancy preferences and their eloquent articulation of the consequences of pregnancy, very few expressed unfulfilled desire for contraception. Adolescents were generally familiar with multiple methods, and were able to communicate where they could get a method; they named public and private health facilities and pharmacies, and some had received condoms from female community health workers. Rather, multiple competing priorities other than avoiding pregnancy emerged from the interviews and FGDs, including assuring future fertility, avoiding side effects and community stigma associated with contraceptive use, and preferences of sexual partners. The apparent incongruence between adolescents’ pregnancy preferences and their contraceptive behaviors was not readily acknowledged by participants in the interviews and FGDs, even when specifically probed by interviewers. In section 3.3, we offer adolescent perspectives on the social influences that contribute to incongruent pregnancy intentions and contraceptive behavior.

### 3.3 Social influences on contraceptive decision-making

#### 3.3.1 Stigma: Sexuality and contraceptive use

Participants frequently used the term “spoiled” to refer to girls who are sexually experienced. While all but a few respondents felt that adolescent contraceptive use was generally more acceptable in their communities than adolescent pregnancy, both were considered stigmatizing and reflected poorly upon families. The minority felt that they would face fewer social consequences if they became pregnant, compared with openly using hormonal contraception. If a girl were discovered to be using a contraceptive method, many participants asserted, she could be labeled as “promiscuous” in the community. An 18 year-old participant, who used condoms consistently to prevent pregnancy but avoided what she called “family planning” explained:

*“They [people in the community] will come to many conclusions [if they see me going for family planning]*, *maybe they will say that I have gone for family planning because I want to have sex*.*”*

—*IDI participant*, *Kisumu*

#### 3.3.2 Community narratives of contraceptive risks

Adolescents spoke of pervasive community narratives of adolescent contraceptive use leading to future infertility or inability to give birth to a healthy baby in the future as a powerful influence on their decisions around contraception. Several participants brought up the term “family planning,” explaining that women who already have a family are the only ones who should plan. An 18 year-old participant reported using condoms only for this reason:

*“People are saying that it [family planning] is not safe…let’s say for example I decide to use family planning and yet I have never gotten pregnant*, *so people say that maybe I can end up being barren…That is the only thing I hear people [say] discouraging use…I think it is because there is no way I can just from nowhere start using family planning*, *I have never been pregnant even for one day*, *so I don’t know whether I can get pregnant or not*. *So you should use after you have gotten pregnant*.*”*

—*IDI participant*, *Kisumu*

Some respondents reported not believing these widespread messages about family planning and infertility, citing examples of people they knew who had conceived after using a method. Others expressed uncertainty about what messaging to believe, but perceived family planning use prior to having a child as the lesser of two evils when compared with adolescent pregnancy:

*“If you are a good girl then you just take care of yourself [abstain from sex]*, *you don’t use family planning until you give birth one day…but nowadays because children start having sex early*, *it forces them to use family planning so that it can help them finish school…it is good if a girl…[uses family planning] until she finishes school then she can become pregnant…It is not a bad idea because for her she is securing her future*.*”*

—*IDI participant*, *Siaya*, *age 14*

Concerns about contraceptive side effects figured prominently into adolescents’ perceptions of their need for contraception. Changes in menstruation and body weight, as well as abdominal pain and reduced sexual urge were frequently mentioned, but were considered less troubling than the risk of infertility. Respondents also alluded to family planning stigma among adults and other adolescents in their communities, primarily associating use with sexual promiscuity and the seeking out of sexual pleasure among girls and young women. As one young woman put it,

*“I have heard that family planning if it doesn’t suit you it can destroy your body…that it causes infertility…They say that family planning is not so good among young girls*. *Because some people once they are put family planning*, *so they just start becoming promiscuous*.*”*

—*IDI participant*, *Siaya*, *age 17*

When participants were asked which specific people in their lives influenced their decisions to use or not use a family planning method, they most frequently named their mothers and boyfriends. Most described their mothers’ advice or directive *against* using contraception other than condoms, usually due to concerns about effects on girls’ health or future fertility:

*“…Sometimes maybe we are sitting in the house*, *our mother usually advises us to abstain from sex and not go for family planning citing that family planning spoils young girls*. *She normally advises us*, *especially me*, *she really advises me because I am the youngest in our house that I should not start using those things [FP methods] now*. *I should not even think of using them…If I start using now*, *in the future when I will want to have children it will be very hard for me to conceive*…*”*

—*IDI participant*, *Siaya*, *age 15*

Mothers had a high level of sway over their daughters’ beliefs about contraception and their comfort accessing it. Condoms were not widely considered a family planning method among participants, however, and a young woman makes this distinction clear:

*“…For me*, *I can get advice from my mother*. *If I have to go for the family planning that is put on the arms [injection*, *implant] then I have to tell her because maybe this thing might bring complications*, *but for condom*, *I can just go without telling her–even if she will realize later*. *If it’s about use of condoms then it is [up to] me to decide*.*”*

—*FGD participant*, *Kisumu*, *age 19*

Female peers were main sources of influence on participants’ sexual behavior, as well as information about contraceptive methods. Peers often propagated community narratives about contraceptive risks. A 14 year-old participant explained:

*“There is a woman I used to live with a long time ago*, *she is the one who told me that family planning is bad*…*that if you used family planning and you are a girl who has never had a child*, *then getting pregnant might be a bit difficult…When she told me like that I went and shared with my girlfriend and she told me that…she cannot use [family planning] because she has also heard that family planning is bad…*.*”*

—*IDI participant*, *Siaya*

Seeing peers get pregnant at school was particularly powerful for some adolescents, who often learned more about contraception from classmates than from parents:

*“Because maybe you are already spoiled [sexually experienced]*, *and you know pregnancy never knocks at the door*, *it is like HIV*, *so that [school] is where we learned about family planning*, *because your parent can never advise you to go for family planning…Those whose parents were refusing that they go for family planning…could just sneak to the hospital*.*”*

—*IDI participant*, *Kisumu*, *age 17*

#### 3.3.3 Power in sexual relationships

While the median age of first sex among sexually active participants was 15 years, the majority of respondents reported that typical sexual debut in the community was between 10–14. Adolescents named peer and partner pressure as primary influences on sexual debut, and most discussed the need for financial support or material gifts from male partners, usually older adolescents or men, in order to have sex with them. A 17-year-old participant who reported first sex at age 13 described how she saw her peers acquiring food, clothing, and other material goods from sexual partners. Her own lack of these items motivated her to start having sex:

*“…maybe you see your friend has a nice dress and you don’t have*, *so you know that boy will tell you that he is going to buy for you*. *So that is the reason she will say…let me go have sex with him so that I can get even inner wears [underwear]*.

—*IDI participant*, *Kisumu*

She went on to talk about her first sexual partner, with whom she said she would not have had sex had he not bought her feminine pads among other things: “*There is no need of someone to feel sweet on your body without helping you*.*”*

Sexual partners both encouraged and discouraged contraceptive use. Some participants felt they were urged to access family planning by boyfriends to avoid the embarrassment of “impregnating” a young girl, or to stop using condoms. Others described pressure from boyfriends to stop using their method due to concerns around future fertility. Respondents described various dynamics around negotiating condom use, but most stated that boys put pressure on girls in general to have unprotected sex:

*“Most of them [girls] may want to use it [condoms] but now the boyfriend might refuse*, *and since she believes that she loves her boyfriend*, *she will just end up following what he says…[Boys] will say that you cannot eat [a] sweet with the cover [on]*.*”*

—*IDI participant*, *Siaya*, *age 17*

Despite the diverse factors and experiences influencing their contraceptive choices, some adolescents expressed awareness of agency over their decisions to prevent pregnancy. A participant who had been sexually active since 14 recalled how a partner told her that condoms affected him negatively and refused to use condoms:

*“Long time ago I used to listen to him when he told me like that*, *but nowadays since I knew how this world is*, *if I have a man*, *I tell him we go for testing and he refuses*, *and I also tell him we use protection and he refuses*. *I just tell him my dear*, *walk with Jesus and you also let me walk with Jesus…(laughter)… because that is someone who doesn’t wish well for you*, *there is something he wants*, *he wants to spoil your future*.*”*

—*IDI participant*, *Kisumu*, *age 19*

When asked who or what influences her decision to use contraception, another young woman who reported using condoms answered:

*“My future…Because if I don’t give birth now I will finish school and I will have a better future and if I give birth now I will drop out of school and I wouldn’t have a good future*.*”*

—*IDI participant*, *Siaya*, *age 18*

## 4. Discussion

Among the 86 adolescents who participated in IDIs and FGDs in this community-based study, we elicited a strong preference to delay pregnancy that was often uncoupled from a desire for contraception. While the majority of participants would be considered to have an “unmet need” for contraception based on the indicator’s definition, their perception of contraceptive need and ultimately their contraceptive decisions were shaped by various factors and priorities within their social contexts. These factors are illustrated in the conceptual model (Fig 2). Informed by social norms and future life aspirations, participants viewed pregnancy in adolescence prior to relative financial independence as an unacceptable outcome. They then had to navigate interrelated “layers” of social messaging that ultimately influenced their contraceptive decision-making and ability to prevent pregnancy. Each “layer” had differing levels of influence on each adolescent. For example, for some, perceived risks of hormonal contraception causing infertility and stigma associated with contraceptive use outweighed pregnancy concerns and motivated a desire for condom use, while unequal power with sexual partners and need for financial support further influenced ability to prevent pregnancy using condoms.

The curved arrows in the model represent the ability of adolescents who desire to delay pregnancy to traverse social norms and narratives, stigma, and unequal power dynamics that may intervene in their ability to prevent pregnancy. Use of a contraceptive method is a primary way that adolescents may exercise agency to prevent pregnancy, and several participants clearly articulated self-efficacy in demanding condom use or using contraception despite dominant social influence. Pulerwitz et al. propose a conceptual framework articulating the relationship between social norms and adolescent SRH that features social and gender norms as a central influence on health outcomes for adolescents [33]. The concept that power can be demonstrated in decisions to “adhere to (or not adhere to) social norms” [33] is relevant to our analysis. Contraceptive use was generally viewed as a transgression of social norms among our participants, but some adolescents made the decision to use a method in spite of social pressures. Furthermore, adolescents’ agentic actions around contraceptive decision-making may not take the form of bold steps [28], but rather smaller and more socially-acceptable steps such as avoiding sex or inconsistent condom use. For example, since condoms are widely considered a mode of HIV prevention rather than a contraceptive method by adolescents, condom use carries less contraceptive stigma than hormonal contraceptive methods, which are known more widely as “family planning.”

While participants frequently reported elements of transactional sexual relationships, few adolescents reported other forms of sexual coercion, such as age-disparate relationships, or sexual assault. We discussed sexual coercion in the interviews, but our study was not focused on examining experiences of coercion and violence in detail. Studies suggest that many Kenyan adolescents have limited interpersonal power in sexual relationships [34], and approximately 20% of Kenyan girls and young women aged 15–24 have experienced sexual violence from an intimate partner in the prior year [35]. Additional research examining how a history of violence affects adolescent contraceptive decision-making is needed.

Stigma emerged as a powerful source of adolescent influence in the present study, consistent with existing research [14], including a qualitative study of postpartum Kenyan adolescents from the same geographic region [17]. “Good girls” abstained from sex, didn’t use contraception, and didn’t get pregnant. The relevant stigmas can be conceptually tied together by participants’ use of the word “spoiled,” directly translated from Luo. Adolescents referred to themselves and peers who had begun sexual activity or were seen as promiscuous as “spoiled,” futures were “spoiled” by adolescent pregnancy, and contraceptive use could “spoil” one’s eggs and make one unable to have children in the future. Indeed, sexually-active adolescents risk experiencing stigma regardless of whether they actively prevent pregnancy through contraceptive use or become pregnant during adolescence. Understanding this context of multidimensional stigma may help to explain adolescents’ apparently incongruent pregnancy preferences and contraceptive behaviors. Our study focused on contextual rather than on health systems factors influencing contraceptive decision-making, which limits our data’s representation of stigma experienced by adolescents in the health care setting. However, prior qualitative and mystery-client studies describe provider bias towards sub-Saharan African adolescents, leading to restriction in method choice due to provider beliefs [36] as well as experiences of shame, embarrassment, and loss of confidentiality among adolescents seeking contraception [37].

Our data elicited little diversity in participants’ current pregnancy preferences: they reported that an unintended pregnancy would be unacceptable. Adolescent pregnancy is common in Kenya: among women aged 20–24 in 2014, nearly 25% of women had given birth by age 18, and 46.8% by the end of the 19^th^ year. Among 15–19 year-olds, 18% were pregnant or had already give birth [38]. Given that context, we anticipated a wider spectrum of positive, ambivalent, or indifferent perspectives on pregnancy “now.” Studies from the United States among young women advocate that unintended pregnancy is not a universally poor outcome; many unplanned pregnancies are welcomed and acceptable [39], and social context, including limited financial resources and social status, may make pregnancy planning less relevant for some [40]. The present study’s narrow scope of pregnancy preferences likely reflects its young, 15–19 year-old population, and the exclusion of married and parous adolescents; it is also important to note that participants viewed future pregnancy extremely favorably. Interestingly, our findings are consistent with a recent quantitative study of ambivalence in pregnancy intentions in Kenya, where the youngest age group of women (age 15–24) were the least likely to express ambivalence [41]. Social desirability bias may have influenced some adolescents’ responses, especially in the FGDs, though many adolescents freely shared other behaviors and experiences that would be considered contrary to social norms. Additionally, as parental consent was required for participants under age 18, adolescents who did not feel comfortable involving their parents, perhaps due to parents’ more conservative attitudes towards sexuality or contraception, may have avoided sampling. However, only 3 adolescents who were otherwise eligible for the study declined due to lack of parental availability, and no parent declined to provide permission. Quotes from IDI participants are primarily used in the results, as they tended to be more concise than the longer FGD conversations, but thematic elements were similar between data sources. Our study was designed to take advantage of the social nature of FGDs and anticipated that that the way peers conversed about the topics may uncover new insights. However, we did not find that the FGDs promoted interaction that elicited different perspectives than the IDIs. While our team thought carefully about word choice when translating interview questions from English into Luo and Kiswahili, there may have been challenges with managing the connotations and translation of various descriptors such as “unplanned” and “unintended.” Despite translation quality control measures, it is possible that some meaning and nuance around pregnancy preferences were lost in translation.

This study’s conceptual framing distinguishes it from other studies assessing adolescent perspectives on contraceptive use. We explored norms and preferences around adolescent sexuality, pregnancy, contraception, and abortion to contextualize adolescent contraceptive behaviors and gain insight into their contraceptive decision-making. This approach allowed us to closely examine the incongruence between adolescent reproductive desires and contraceptive behavior through the lens of “spoiled” girls’ narratives, beliefs, and life experiences. With this incongruence in mind, individual- and community-level interventions to prevent undesired pregnancy should focus on supporting adolescent decision-making agency rather than increasing contraceptive prevalence, with the goal of better aligning reproductive preferences and health behaviors to achieve those preferences. Such efforts might support adolescents in navigating various “layers” of social norms, such as providing adolescent-centered contraceptive counseling and decisional support, addressing misperceptions, and allowing adolescents to access contraception with peers or in locations not associated solely with contraception such as pharmacies [42, 43] and other community-based venues. Clinical interventions must acknowledge and address pervasive social messaging linking contraceptive use with future infertility. Furthermore, efforts to engage adolescents and young adults, including boys and men, in shifting harmful social and gender norms at the level of communities [44, 45] are also critical to enabling social environments for adolescent SRH care-seeking and healthy behaviors.

The adolescent voices represented in our study support the perspective that contraceptive prevalence rate and unmet need for contraception are useful indicators to compare over time, but are not person-centered outcome measures [46] for programs and interventions aiming to improve adolescent SRH. Indeed, our findings and conceptual model identify domains that should be considered in such adolescent-specific measures of reproductive desires and preferences. Senderowicz calls attention to the mismatch between stated rights-based global family planning goals and the metrics currently used to evaluate family planning programs [21]. Contraceptive autonomy, defined as “the factors that need to be in place in order for a person to decide for themselves what they want in regards to contraceptive use, and then to realize that decision,” is proposed as a novel family planning indicator [21]. Measures such as contraceptive autonomy, reproductive autonomy [47], and sexual and reproductive empowerment [48] should be tailored for adolescents and used to guide programs and funding priorities. Finally, contraceptive access and the ability to prevent pregnancy with contraception when desired are clearly critical to efforts to improve adolescent SRH outcomes. However, as was poignantly expressed in our participants’ stories, a focus on prevention of undesired pregnancy alone is inadequate and unrealistic: along with contraceptive services, quality maternity care, safe abortion services, and post-abortion harm reduction interventions are urgently needed to reduce maternal morbidity and mortality and improve quality of life among adolescents living in Sub-Saharan Africa.

## Supporting information

S1 File In-depth interview guide, English. (PDF)

S2 File In-depth interview guide, Dholuo. (PDF)

S3 File Focus group discussion guide, English. (PDF)

S4 File Focus group discussion guide, Dholuo. (PDF)
