# Supplementary material for: “Spoiled” girls: Understanding social influences on adolescent contraceptive decision-making in Kenya
Source: PLoS One. 2021 Aug 12;16(8):e0255954. doi: 10.1371/journal.pone.0255954 (PMC8360567; doi:10.1371/journal.pone.0255954)
Supplement: S1 File — (PDF) [file pone.0255954.s001.pdf]

S1 Supporting Information

Interview Guide: Adolescents

The questions below will be used as a guide for the conversation with adolescent women.
Questions may be rephrased and probed in various ways.

Primary interview domains:

- Community narratives about adolescent sexual debut, pregnancy, and family planning
- Concept of "unplanned" or "unintended" pregnancy and potential consequences of unplanned pregnancy
- Communication about sexuality, fertility, and family planning with peers, family, authority figures
- Agency in family planning (non)decision-making
- Social influence with respect to sexual relationships, pregnancy/pregnancy prevention
- Perspectives on family planning needs and challenges among adolescents
- Stigma related to family planning and abortion

Thank you for being willing to speak with me about your thoughts and experiences. I'd like to start by asking you about people who are important friends and support people in your life.

Social network

Who are the most important support people in your life? [Friends and family members? Why are they important?]

Tell me about your friends.

- How old are your friends in relation to you? [e.g. older (how much), younger, about the same age] Why do you think that is?
- Think about your closest friend: how old is s(he)? How often do you see her (him)?
    - What do you usually talk about? [e.g. school, work, relationships, current events, money, future plans]
    - Do you live near each other? Talk on the phone?

How do you spend your time outside of your duties at home / school / work? With whom do you spend time?

Now I'd like to ask you a bit more about who you talk to about sensitive topics. I want to remind you that what we talk about here will be kept confidential—that is, we will not discuss what you say with anyone, including your parents.

Who do you talk about sex with?

- How does sex comes up in conversation, and how often? Why do you feel comfortable talking about it with [person]?
- [If says no one] Why do you think you don't talk about sex with people close to you? Has anyone ever talked to you about sex?

What about family planning? Who do you talk to/have you talked to about family planning?

- On which occasions has family planning come up in conversations with family members? Friends? Can you tell me about those conversations?
    - Which methods of family planning have you discussed with [person/people]?

Now I'll be asking you some questions about your thoughts about pregnancy and pregnancy prevention.

Pregnancy intendedness
What are your thoughts about pregnancy?

- Have you given any thought to becoming pregnant now? What about in the future? What are your thoughts about becoming pregnant now/in the future?
- When do you think you might want to become pregnant?
    - Why would that be an ideal time? [e.g. age, marriage, proving fertility, financial security, education complete]

If you became pregnant now, how do you think you would react?

- Your parents/family? Your friends?
- How do you think having a child now would affect your life now? What about in the future?
- Who in your life has become pregnant around your age? What was her experience? What did you think when she became pregnant?

What do you think about the idea of planning a pregnancy, versus when a pregnancy "just happens"?

- Can you think of someone you know who had a planned pregnancy? What was her experience?
- Can you think of someone you know who had a surprise pregnancy or a pregnancy that "just happened"? What was her experience?
- Who in your life has become pregnant at a time she did not want to become pregnant? Could you tell me more about her story? What happened with the pregnancy, and how did she cope?
- Tell me what you know about abortion? Without using names, could you tell me about someone who has tried to end a pregnancy/have an abortion. What happened? Anyone else? What do you/people in your community think about abortion?
- If a friend told you she wanted to have an abortion, how would you react? Would you know where she should go?

In your community, what is the message (community perception) that people your age get about having sex?

- When do people your age usually start having sex? Why do you think this is?
- What do girls your age think about using condoms? Boys your age?
- How did you decide to start having sex? Did anyone influence or pressure you to have sex, or give you money or gifts in exchange for sex? *[Probe for anecdotes and personal experiences]*

Family planning

What do people in your community think about people your age using a family planning method? What about your parents or other adult family members?

- Why do you think they have those beliefs?
- What do you think is more accepted: someone your age becoming pregnant, or someone your age using family planning? Why?

What are your sources of information about family planning? *[e.g. family, friends, teachers, spiritual leaders, chemist, health care providers]*

Where do people your age get family planning? Why?

What do you think people your age think about using family planning?

- What are some potential benefits of using family planning? Risks?
    - What do people your age say about family planning side effects? What about having children in the future after using family planning?

- Which methods are most popular among people your age? Why?
    - *Probe: condoms, withdrawal, emergency contraception, depo, pills, implant, IUCD, other*
- What concerns do girls your age have about family planning?
- What concerns do boys your age have about family planning?

Tell me about your family planning experience. Have you ever used a method?

- [If yes] Which one(s)? What was your experience like?
    - How did you decide to use the method? [after vs before sexual debut]
    - Was your partner aware? [If so] What was his reaction?
    - What concerns did you have about using [method]?
    - How did you access [method]?
- What about condoms or withdrawal? *(if not already mentioned)*
- [if no] How did you decide NOT to use a method?
    - Did you have any concerns about becoming pregnant? *Probe: Tell me more about that.*
    - What concerns did you have about using a family planning method? What made up your mind not to use it? *[Alternatively, if participant did not really think about it, explore her risk perception around pregnancy]*

What do you know about the E-pill? [*Probe for anecdotes and personal experience vs community experience]*

What other stories or information have you heard in the community about family planning? Which stories/information do you think are true/untrue? [*Probe for anecdotes*]

Some girls your age feel like they should give birth before starting family planning, to ensure they can have children. What do you think about this? [*Probe: why?*]

What would it be like as someone your age to go to the health center for family planning? [If has used FP] Tell me about the experience of going to the clinic to get family planning.

- What was the interaction with the nurse like? (What would the interaction with the nurse be like?)
- Do you think people in the community would think about you differently if they found out you were using family planning? Why?

Who do you think influences you the most in your decisions about family planning? *[e.g. Family, friends, teachers, spiritual leaders, health care providers]* Why do you think that is? Could you give an example of how [person/people] have influenced you?

It is fairly common for girls your age to become pregnant when they do not yet feel ready for a baby. How do you think this could be prevented?

- How do you think family planning could be easier to access for you? People your age?
- What would you recommend in terms of engaging people your age in the community around reproductive health—that is, health related to sex, having children, or preventing pregnancy?

Do you have any other thoughts you would like to add, or a story/experience you haven't yet shared that you think is important?

Thank you very much for your time and willingness to share your thoughts. Everything you shared today will be held in the highest confidence.
