# Supplementary material for: “Spoiled” girls: Understanding social influences on adolescent contraceptive decision-making in Kenya
Source: PLoS One. 2021 Aug 12;16(8):e0255954. doi: 10.1371/journal.pone.0255954 (PMC8360567; doi:10.1371/journal.pone.0255954)
Supplement: S2 File — (PDF) [file pone.0255954.s002.pdf]

Oboke ma tayo penjo: Rowere

Penjo mantie piny kae gi ibiro tiyo godo kaka penjo matayo mbaka gi mine ma rowere.
Penjogi inyalo penj ka iloko kendo inuoyo penjo gi e yore ma opogore opogore.

Primary interview domains:
- Community narratives about adolescent sexual debut, pregnancy, and family planning
- Concept of "unplanned" or "unintended" pregnancy and potential consequences of unplanned pregnancy
- Communication about sexuality, fertility, and family planning with peers, family, authority figures
- Agency in family planning (non)decision-making
- Social influence with respect to sexual relationships, pregnancy/pregnancy prevention
- Perspectives on family planning needs and challenges among adolescents
- Stigma related to family planning and abortion
- Perspectives on community-level strategies to reduce barriers to SRH services for adolescents

Erokamano kuom yie loso koda kuom pachi kod lony mari. Daher mar chako ka apenji kuom ji ma omokoni e ngimani, osiepe kod jogo ma miyi siro e ngimani.

Tudruok mar oganda

Gin ng'ano gini ma omokoni ma miyi siro e ngimani – osiepe kod anyuala? Ang'o ma omiyo gin ema gimokoni?

Nyisa kuom osiepeni.
- Osiepeni ka ipimo kodi gin johigni adi? [e.g. gihingi (marom nade), ihingogi, un mbese] Ang'o ma omiyo iparo ni en kamano?
- Par ane kuom asiepni ma in go machiegni ahinya: en ja higni adi? Inenega machiegni marom nade?
    - En ang'o ma ng'enyne ujawacho kode? [e.g. skul, tich, tudruok, gik matimore sani, pesa, chenro mag ndalo mabiro]
    - Bende udak machiegni? Uloso e simu?

Ere kaka ikalo ga secheni ka opogore gi tijeni mag dala/skul/tich? Ibudho ga gi ng'a?

Koro adwa penji mang'eny matin kuom joma iwuoyo ga godo kuom weche ma opondo. Adwaro paroni ni gimoro amora ma wawacho kae ibiro kan maopondo – tiendeni, ok wa bi lose kuom gik ma wawuoye gi ng'ato ang'ata, koriwo nyaka jonyuolni.

En ng'a ma iwuoyoga godo kuom weche mag bedo e achiel?
- Ere kaka weche mag bedo e achiel biro ga e mbakau, kod ding'eny marom nade? Ere gima omiyo in ga thuolo wuoyo kuom wechegi gi [Ng'atno]?
- [If says no one] Ere gima omiyo ok iwuoga kuom weche mag bedo e achiel gi jok machiegni kodi? Bende ng'ato osewuoyo ga kodi kuom weche mag bedo e achiel?

To yore mag komo nyuol? En ga ma iwuoyo ga godo/ ma isewuoyo godo kuom komo nyuol?
- En karang'o mane mbaka owuok ewi komo nyuol gi anyulani? Osiepe? Bende inyalo nyisa kuom mbekni go?
    - Gin yore mage mag komo nyuol ma usewuoye gi [ng'atno/ jogo]?

Koro adhi penji penjo moko kuom pachi ewi mako ich kod geng'o mako ich.

Chano mako ich

Pachi en ang'o kuom mako ich?
- Bende iseparo mar mako ich sani? To e ndalo mabiro? Pachi en ang'o kuom mako ich sani kata e ndalo mabiro?

- Iparo ni inyalo dwaro mako ich karang'o?
  - Ang'o ma omiyo kindeno ber? [e.g. hiki, osekendi, nyiso ni inyalo mako ich, bedo gi omuom moromo, tieko somo]

Ka imako ich sani, iparo ni inyalo kawe nade?
- Jonyuolni/anyuolani? Osiepeni?
- Ere kaka bedo gi nyathi sani nyalo chacho ngimani sani? To e ndalo mabiro?
- Ng'a e ngimani ma osemako ich ma mbasni? Lony ne nene chalo nade? Ne iparo ang'o kane omako ich?

Iparo ang'o kuom paro mar chano mako ich, kipimo gi mako ich ma ipoyo apoya?
- Bende inyalo paro ng'ato ma ing'eyo ma ne omako ich ka ochano? Lonyne nene chalo nade?
- Bende inyalo paro ng'ato ma ing'eyo mane omako ich apoya kata ich ma odonjo apoya? Lonyne nene chalo nade?
- En ng'ano e ngimani ma ne omako ich e kinde ma ne ok odwar mako ich? Bende inyalo nyisa matut kuom sigande? Ang'o mane otimre gi ijno, to ere kaka ne otimo?
- Nyisa kuom ngatma osetemo golo ich? En ang'o mane otimore? Ng'ama chielo? En ang'o ma in/ji e ogandau paro kuom golo ich?
- Ka osiepni onyisi ni odwaro golo ich, inyalo kawe nade? Bende inyalo ng'eyo kama onego odhiye?

E ogandau, en ang'o ma rowere ka in ong'eyo kuom weche mag bedo e achiel?
- En karang'o ma rowere ka in chako ga bedo e achiel? Iparo ni en kamano nikech ang'o?
- En ang'o ma nyiri ma mbeseni paroga kuom tiyo gi rabo yunga? To yawuoyi ma mbeseni?
- Ere kaka ne iyiero mar chako bedo e achiel? Bende ng'ato ne ooli e bedo e achiel? *[Probe for anecdotes and personal experiences]*

Komo nyuol

En ang'o ma ji e ogandau paro kuom ji marom kodi ma tiyo gi yore mag komo nyuol? To jonyuolni kata jomadongo e anyualani?
- Ango ma omiyo iparo ni giparo kamano?
- Ang'o ma iparo ni ji oyiego ahinya: ng'at marom kodi ka omako ich, kata ng'at marom kodi tiyo gi yore mag komo nyuol? Nang'o?

Ere kama iyudogae weche kuom komo nyuol? *[e.g. anyuolani, osiepe, jopuonj, jotend dini, jochiw thieth]*

Iparo ni ji ma mbeseni paro ang'o kuom tiyo gi yore mag komo nyuol?

- Ber mar tiyo gi yore mag komo nyuol gin ang'o gini? Rach?
  - En ang'o ma ji ma mbeseni wacho ga kuom rach ma otudore gi tiyo gi yore mag komo nyuol? To kuom bedo gi nyithindo e ndalo mabiro ka itiyo gi yore mag komo nyuol?

- Gin yore mage ma ji ma mbeseni ohero ahinya? Nang'o?
  - *Probe: Raboyunga, wuodho yunga kapok pi nyodo owuok, andila mar geng'o ich mapiyo, sindan, andila mag geng'o ich, mar bat, koil, mamoko*
- Gin chich mage ma nyiri ma mbeseni nigo kuom yore mag komo nyuol?
- Gin chich mage ma yawuoyi ma mbeseni ni godo kuom yore mag komo nyuol?

Nyisa kuom lony ni ewi yore mag komo nyuol. Bende isega tiyo gi yor komo nyuol?

- [Ka ee] Mane/mage? Lony ni nene chalo nade?

- Ere kaka ne iyiero tiyo gi yor komo nyuol no? [bange kipimo gi kapok ne ichako bedo e achiel]
  - Bende jaherani ne ong'eyo? [ka kamano] Ne okawe nang'o?
  - En chich mane ma ne in godo kuom tiyo gi yor komo nyuol?
  - Ere kaka ne iyudo yor komo nyuol no?
- [Ka ooyo] To rabo yunga kata wuodho yunga kapok pi nyodo owuok?
- [Ka ooyo] Ere kaka ne iyiero mar wero tiyo gi yor komo nyuol?
  - Bende ne in gi chich moro amora kuom mako ich? *Probe: Nyisa matut kuom ma.*
  - Gin chich mage mane in godo kuom tiyo gi yore mag komo nyuol? En ang'o mane okonyi yiero ni ok iti kode? *[Alternatively, if participant did not really think about it, explore her risk perception around pregnancy]*

En ang'o ma ing'eyo kuom andila mar geng'o ich piyo? [*Probe for anecdotes and personal experience vs community experience]*

Gin sigana mage mamoko ma isewinjo e oganda kuom yore mag komo nyuol? Sigana mage ma iparo ni gin adiera/ miriambo? [*Probe for anecdotes*]

Nyiri moko ma mbeseni nenoga ni onego gi nyuol mondi kapok gichako tiyo gi yore mag komo nyuol, mondo gine ni ginyalo yudo nyithindo. In iparo ang'o kuom ma? [*Probe: Nang'o?*]

Nyalo bedo machal nadi ka ng'at ma mbasni odhi e kar thieth ne yore mag komo nyuol? [If has used FP] Nyisa kuom lony mar dhi e klinik yudo yor komo nyuol.

- Tudruok ni gi sista ne chal nade? (Tudruok gi sista nyalo chalo nade?)
- Iparo ni ji ma nitie e ogandau nyalo paro kuomi ma opogore ka gifwenyo ni itiyo gi yore mag komo nyuol? Nang'o?

En ng'a ma iparo ni osesieyi ahinya e yieroni kuom yore mag komo nyuol? *[e g. Anyuolani, osiepeni, jopuonj, jotend dini, jochiw thieth] Ang'o ma omiyo iparo ni en kamano*? Bende inyalo chiwo ranyisi mar kaka [ngato/ji] ne osieyi?

En gima timore mang'eny mondo nyiri mambeseni omak ich kapok giyikore mar yudo nyathi. Ere kaka iparo ni ma inyalo geng?

- Iparo ni ere kaka yudo yore mag komo nyuol nyalo bedo mayot ne in? Joma mbeseni?
- En rieko mane ma inyalo ng'ado ewi tudruok gi mbeseni e oganda kuom yore mag ngima ma otudore gi yor nyuol – tiendeni, ngima ma otudore gi bedo e achiel, bedo gi nyithindo, kata geng'o mako ich?

Bende in gi paro mamoko ma diher medo, kata sigana/lony ma pok inyisa ma iparo ni ber mondo ang'e?

Ero kamano ahinya kuom thuoloni kod yie mari mar chiwo pachi. Gimoro amora ma ne iwacho kawuono ibiro kan ma opondo ahinya.
