# Supplementary material for: “Spoiled” girls: Understanding social influences on adolescent contraceptive decision-making in Kenya
Source: PLoS One. 2021 Aug 12;16(8):e0255954. doi: 10.1371/journal.pone.0255954 (PMC8360567; doi:10.1371/journal.pone.0255954)
Supplement: S4 File — (PDF) [file pone.0255954.s004.pdf]

S4 Supporting Information

Oboke ma tayo Twak mar Kidieny (FGD): Rowere

Penjo mantie piny kae ibiro tiyo godo kaka gima tayo twak mar kidieny gi rowere mamine. Penjo inyalo penj ka iloko kendo inuoyogi kipenjogi matut e yore ma opogore opogore.

Primary FGD domains:

- Community narratives about adolescent sexual debut, pregnancy, and family planning
- Concept of "unplanned" or "unintended" pregnancy and potential consequences of unplanned pregnancy
- Communication about sexuality, fertility, and family planning with peers, family, authority figures
- Agency in family planning (non)decision-making
- Social influence with respect to sexual relationships, pregnancy/pregnancy prevention
- Perspectives on family planning needs and challenges among adolescents
- Stigma related to family planning and abortion
- Perspectives on community-level strategies to reduce barriers to SRH services for adolescents

Erokamano uru kuom biro kawuono, kendo kuom yie chiwruok e twak mar kidienyni. Watemo puonjre kuom mine matindo ma mbeseu ewi ng'at ma nyalo sieou mogik e timo yiero ma moluwore gi ngima mar yor nyuol—Ngima ma otudore gi bedo e achiel, ich, kod yore mag komo nyuol. Dwaher bende ng'eyo lony maru kod yierou ma oluwore gi ngima mar yor nyuol, mondo wayud yore ma wanyalo siro godo mine matindo mondo obed mangima.

Pachu dute omoko nua, kendo onge duoko maber kata marach. Wakwayou mondo ulos ng'ato achiel achiel ka nyalore mondo wawinj gima ng'ato ka ng'ato wacho. Kendo, wakwayo mondo ukan gik ma owach eiye kae kawuono mopondo. Tiendeni, kik iwuo gi jok mamoko mane onge kae kawuono kuom gik ma watwake. Penjo moro amora?

Daher mar chako ka apenjou kuom ji ma omokonu ma gin osiepeu kod ji ma miyou siro e ngimau.

Tudruok mar oganda

Gin ng'ano gini ma omokoni ma miyi siro ahinya e ngimani? [*Probe: Osiepe kod anyuolau*? Ang'o ma omiyo gin ema gimokoni?

Nyisa kuom osiepeni.

- Osiepeni ka ipimo kodi gin johigni adi? [e.g. gihingi (marom nade), ihingogi, un mbese] Ang'o ma omiyo iparo ni en kamano?
- Par ane kuom asiepni ma in go machiegni ahinya: en ja higni adi? Inenega machiegni marom nade?
    - En ang'o ma ng'enyne ujawacho kode? [e.g. skul, tich, tudruok, gik matimore sani, pesa, chenro mag ndalo mabiro]

Ere kaka ikalo ga secheni oko mar tijeni mag dala/skul/tich? Ibudho ga gi ng'a?

Koro adwa penji mang'eny matin kuom joma iwuoyo ga godo ewi weche ma opondo. Adwaro paroni ni wasewinjore ni twak wa ni dhi bedo malingling, kendo ok wadhi nyiso joma moko ma onge e ot ka gima wa twak e.

En ng'a ma iwuoyoga godo kuom weche mag bedo e achiel?

- Ere kaka weche mag bedo e achiel biro ga e mbakau, kod ding'eny marom nade? Ere gima omiyo in ga thuolo wuoyo kuom wechegi gi [Ng'atno]?

- [If says no one] Ere gima omiyo ok iwuoga kuom weche mag bedo e achiel gi jok machiegni kodi? Bende ng'ato osewuoyo ga kodi kuom weche mag bedo e achiel?

To yore mag komo nyuol? En ga ma iwuoyo ga godo/ ma isewuoyo godo kuom yore mag komo nyuol?

- En karang'o mane mbaka owuok ewi yore mag komo nyuol gi anyulani? Osiepe? Bende inyalo nyisa kuom mbekni go?
    - Gin yore mage mag komo nyuol ma usewuoye gi [ng'atno/ jogo]?

Koro adhi penji penjo moko kuom pachi ewi mako ich kod geng'o mako ich.

Chano mako ich

En kinde mage manyalo bedo ni maber mar mako ich mokuongo? Nango?

- Ango ma omiyo [example from group] nyalo bedo ni maber? [hiki, kend, nyiso ni inyalo mako ich, bedo gi pesa moromo, tieko skul]

En ango ma ogandau paroga kuom ji ma mbeseni ma mako ich? Nango? [*Probe for anecdotes*]

- To jonyuolni/anyuolani? Osiepeni?
- Parane kuom ng'ama mbasni mane omako ich. Ere kaka mako ich nene ochacho ngimane? Lony ne ne chalo nade?

Iparo ang'o kuom paro mar chano mako ich, kipimo gi mako ich ma ipoyo apoya?

- Bende inyalo paro ng'ato ma ing'eyo ma ne omako ich ka ochano? Lonyne nene chalo nade?
- Bende inyalo paro ng'ato ma ing'eyo mane omako ich apoya kata ich ma odonjo apoya? Lonyne nene chalo nade?
- En ng'ano e ngimani ma ne omako ich e kinde ma ne ok odwar mako ich? Bende inyalo nyisa matut kuom sigande? Ang'o mane otimre gi ijno, to ere kaka ne otimo?
- Nyisa kuom ngatma osetemo golo ich? En ang'o mane otimore? Ng'ama chielo? En ang'o ma in/ji e ogandau paro kuom golo ich?
- Ka osiepni onyisi ni odwaro golo ich, inyalo kawe nade? Bende inyalo ng'eyo kama onego odhiye?

E ogandau, en ang'o ma rowere ka in ong'eyo kuom weche mag bedo e achiel?

- En kinde mage ma rowere ka in chako ga bedo e achiel? Iparo ni en kamano nikech ang'o?
- En ang'o ma nyiri ma mbeseni paroga kuom tiyo gi rabo yunga? To yawuoyi ma mbeseni?
- Ere kaka ne iyiero mar chako bedo e achiel? Bende ng'ato ne ooli e bedo e achiel? *[Probe for anecdotes and personal experiences]*

Yore mag komo nyuol

En ang'o ma ji e ogandau paro kuom ji marom kodi ma tiyo gi yore mag komo nyuol? To jonyuolni kata jomadongo e anyualani?

- Ango ma omiyo iparo ni giparo kamano?

- Ang'o ma iparo ni ji oyiego ahinya: ng'at marom kodi ka omako ich, kata ng'at marom kodi tiyo gi yore mag komo nyuol? Nang'o?

Ere kama uyudogae weche kuom yore mag komo nyuol? *[e.g. anyuolani, osiepe, jopuonj, jotend dini, jochiw thieth]*

Iparo ni ji ma mbeseni paro ang'o kuom tiyo gi yore mag komo nyuol?

- Ber mar tiyo gi yore mag komo nyuol gin ang'o gini? Rach?
    - En ang'o ma ji ma mbeseni wacho ga kuom rach ma otudore gi tiyo gi yore mag komo nyuol? To kuom bedo gi nyithindo e ndalo mabiro ka itiyo gi yore mag komo nyuol?

- Gin yore mage mag komo nyuol ma ji ma mbeseni ohero ahinya? Nang'o?
    - *Probe: rabo yunga, wuodho yunga kapok pi nyodo owuok, andila mar geng'o ich mapiyo, sindan, andila mar komo nyuol, mar bat, koil, mamoko*

- Gin chich mage ma nyiri ma mbeseu nigo kuom yore mag komo nyuol?
- Gin chich mage ma yawuoyi ma mbeseu ni godo kuom yore mag komo nyuol?

Nyisa kuom lony u ewi yore mag komo nyuol. Yore mage mag komo nyuol ma isegatiyo godo?

- [Ka ee] Mane/mage? Lony ni nene chalo nade?
    - Ere kaka ne iyiero tiyo gi yor komo nyuol no? [bange kipimo gi kapok ne ichako bedo e achiel]
    - Bende jaherani ne ong'eyo? [ka kamano] Ne okawe nang'o?
    - En chich mane ma ne in godo kuom tiyo gi yor komo nyuol?
    - Ere kaka ne iyudo yor komo nyuol no?
- [Ka ooyo] To rabo yunga kata wuodho yunga kapok pi nyodo owuok?
- [Ka ooyo] Ere kaka ne iyiero mar WEYO tiyo gi yor komo nyuol?
    - Bende ne in gi chich moro amora kuom mako ich? *Probe: Nyisa matut kuom ma.*
    - Gin chich mage mane in godo kuom tiyo gi yore mag komo nyuol? En ang'o mane okonyi yiero ni ok iti kode? *[Alternatively, if participant did not really think about it, explore her risk perception around pregnancy]*

En ang'o ma ing'eyo kuom andila mae geng'o ich mapiyol? [*Probe for anecdotes and personal experience vs community experience]*

Gin sigana mage mamoko ma usewinjo e oganda kuom yore mag komo nyuol? Sigana mage ma iparo ni gin adiera/ miriambo? [*Probe for anecdotes*]

Nyiri moko ma mbeseu nenoga ni onego gi nyuol mondi kapok gichako tiyo gi yore mag komo nyuol, mondo gibed gi adiera ni ginyalo nyuolo nyithindo. In iparo ang'o kuom ma? [*Probe: Nang'o?*]

Nyalo bedo machal nade kaka ng'at ma mbasu ka odhi e kar thieth ne yore mag komo nyuol? [ka osetiyo gi yore mag komo nyuol] Nyisa kuom lony mar dhi e klinik yudo yor komo nyuol.

- Tudruok ni gi sista ne chal nade? (Tudruok gi sista nyalo chalo nade?)
- Iparo ni ji ma nitie e ogandau nyalo paro kuomi ma opogore ka gifwenyo ni itiyo gi yore mag komo nyuol? Nang'o?

Gin ng'a gini ma uparo ni sieyou ahinya e yierou kuom yore mag komo nyuol? *[e g. Anyuolani, osiepeni, jopuonj, jotend dini, jochiw thieth]* Ang'o ma omiyo iparo ni en kamano? Bende inyalo chiwo ranyisi mar kaka [ngato/ji] osesieyou?

En gima timore mang'eny ne nyiri ma mbesiu mondo omak ich kapok giyikore mar yudo nyathi. Ere kaka uparo ni ma inyalo geng?

- Iparo ni ere kaka yudo yore mag komo nyuol nyalo bedo mayot ne ji ma mbeseu?
- En rieko mane ma inyalo ng'ado ewi tudruok gi ji ma mbeseu e oganda kuom yore mag ngima ma otudore gi yor nyuol – tiendeni, ngima ma otudore gi bedo e achiel, bedo gi nyithindo, kata geng'o mako ich?

Bende in gi paro mamoko ma diher medo, kata sigana/lony ma pok inyisa ma iparo ni ber mondo ang'e?

Ero kamano ahinya kuom thuoloni kod yie mari mar chiwo pachi. Gimoro amora ma ne iwacho kawuono ibiro kan ma opondo ahinya.
